# Association of the humoral immune response with the inflammatory profile in *Plasmodium vivax* infections in pregnant women

**DOI:** 10.1371/journal.pntd.0012636

**Published:** 2024-11-04

**Authors:** Rodrigo Medeiros de Souza, Maria Inês dos Santos, Laura Cordeiro Gomes, Bruna Beatriz Pedroza de Melo, Erika Paula Machado Separovic, Oscar Murillo, Gerhard Wunderlich, Taane Gregory Clark, Susana Campino, Sabrina Epiphanio, Claudio Romero Farias Marinho, Jamille Gregório Dombrowski

**Affiliations:** 1 Multidisciplinary Center, Federal University of Acre, Acre, Brazil; 2 Department of Parasitology, Institute of Biomedical Sciences, University of São Paulo, São Paulo, Brazil; 3 Faculty of Infectious and Tropical Diseases, London School of Hygiene & Tropical Medicine, London, United Kingdom; 4 Faculty of Epidemiology and Population Health, London School of Hygiene & Tropical Medicine, London, United Kingdom; 5 Department of Clinical and Toxicological Analyses, School of Pharmaceutical Sciences, University of São Paulo, São Paulo, Brazil; CSIR-Indian Institute of Chemical Biology, INDIA

## Abstract

**Background:**

*Plasmodium vivax* infection, when it occurs during pregnancy, has often been associated with serious adverse pregnancy outcomes. However, immunological alterations in pregnancy and their consequences have been little explored. We characterized the humoral immune response in pregnant women exposed to malaria by *P*. *vivax* antigens and its association with the maternal inflammatory profile and poor pregnancy outcomes.

**Methods:**

An observational cohort study in the Brazilian Amazon was conducted between 2013 and 2015. After applying exclusion criteria, 242 mother-child pairs were included in the analysis. Data on maternal infection, gestational outcomes, and inflammatory factors were evaluated in the maternal peripheral plasma. In samples from the first infection, the presence of total IgG and its subclasses in plasma against PvMSP1_19_ protein were also quantified.

**Results:**

Previous exposure to malaria, observed by anti-total IgG antibodies to the PvMSP1_19_ antigen, increased the inflammatory response to infection when the pregnant woman had malaria during pregnancy. IL-6 and IL-10 levels were positively correlated with parasitemia and with total IgG levels; but they were negatively correlated with the gestational age at delivery from *Pv*-infected woman. In multivariate linear regression analyses, IgG 1, 2 and 4 was negatively and positively associated with cytokines IL-6 and IL-10, respectively, in *P*. *vivax-*infection.

**Conclusions:**

An association between the humoral immune response and the peripheral inflammatory cytokine profile with the adverse outcomes in malaria in pregnancy by *P*. *vivax* was observed. Previous exposure to the parasite can influence the IL-6 and IL-10 response, which is associated with increased parasitemia, reduced maternal weight gain and premature delivery.

## Author summary

*Plasmodium vivax* malaria in pregnancy is also associated with several adverse outcomes, even in low-transmission regions such as South America. However, to our knowledge, no study has previously investigated the link between the humoral immune response due to previous exposure to *P*. *vivax* and the immunological profile during pregnancy infection and its association with poor pregnancy outcomes. In this study, after applying exclusion criteria, 242 mother-child pairs were evaluated for different immunological markers of malaria exposure in an endemic area in the Brazilian Amazon. We showed that previous exposure to *P*. *vivax*, through the humoral immune response to the PvMSP1_19_ antigen, can exacerbate the inflammatory response during gestational malaria and is associated with increased parasitemia, reduced maternal weight gain and premature delivery. These findings help to understand the association between anti-PvMSP1_19_ antibodies, considered exposure marker, and the maternal peripheral inflammatory profile throughout pregnancy, bringing important implications for understanding the immunobiology of the disease.

## Introduction

The life cycle of *Plasmodium vivax (Pv)* malaria parasites is complex, with different forms in the vertebrate host, extensive genetic diversity and antigenic polymorphism [1,2]. These characteristics promote evasion of parasite clearance, preventing the establishment of an effective immune response and allowing the parasite to remain alive in the host for extended periods [3]. Furthermore, the inflammatory process induced by the infection involves anti- and pro-inflammatory cytokines, and can cause an immunological imbalance during the gestational period, which has been associated with poor gestational outcomes [4,5].

By convention, the immune response to the parasite has been divided into two phases: pre-erythrocytic, involving sporozoites and tissue schizonts; and erythrocytic or blood stage, which is directed against merozoites and intraerythrocytic parasites [6]. This last phase is considered of greater importance mainly because it is related to the clinical protection of the disease [7].

In recent years, there have been advances in the characterization of antigens of blood forms, and an improved understanding of the immunological mechanisms that are involved in the elimination of the parasite [7]. Among the main antigens of blood forms already characterized and considered as possible vaccine candidates, the surface proteins of merozoites, such as *Merozoite Surface Protein-1* (MSP-1), stand out [6]. This protein is one of the most abundant merozoite proteins and is encoded by a single copy gene [8,9]. MSP-1 has a highly polymorphic portion called Block-2 that seems to be under pressure from the immune response given its association with antibodies and clinical immunity [10–12].

It is known that pregnant women are more susceptible to malaria and its complications [13,14]. The exacerbated inflammatory process generated by the infection and the acquisition of clinical immunity to malaria is dependent on the antibody-mediated response, which is a complex and multifactorial process [15]. Importantly, the development of this protective immunity to malaria is slow and, in order to be maintained, requires constant exposure to antigenic variants, as well as the maturation of the immune system [16]. In infections caused by *P*. *falciparum*, it is known that the different classes and subclasses of antibodies produced are associated with protective immunity [17,18], but little is known about this protection in *P*. *vivax* infections. For this reason, we characterized the humoral immune response to PvMSP1_19_ antigen in pregnant women and its association with the inflammatory profile of infection and poor pregnancy outcomes for both mother and newborn.

## Methods

### Ethics statement

This study was approved by the Ethics Committee from the University of São Paulo (Plataforma Brasil, CAAE n° 90474318.4.0000.5467). All pregnant women or their legal guardians (if minors) provided written informed consent.

### Study design and participants

This study is a secondary analysis of an observational cohort study conducted by our group between 2013 and 2015 in the Amazon Region [19,20]. Socioeconomic, clinical, and obstetric information were recorded, in addition to the collection of peripheral blood throughout the pregnancy. In the present study, after exclusions, 242 women were analyzed (**S1 Fig**). Pregnant women who used alcohol, illicit drugs and/or smoked during pregnancy, with a history of pre-eclampsia, who had other infections including helminthiasis, multiple pregnancies and babies with congenital anomalies were excluded. Those whose gestational age had not been defined by ultrasound, as well as those diagnosed with a mixed infection or by *P*. *falciparum* by molecular biology (RT-PCR), were also excluded. Finally, to avoid bias in the analyzes and interpretations, 44 pregnant women who had only *P*. *vivax* infection, but who had not been followed up since the first infection, were excluded (**S1 Fig**). All clinical procedures and pregnancy outcomes are described elsewhere [19,20].

### Expression and purification of the recombinant protein

The recombinant antigen PvMSP1_19_ was used to evaluate the immune response of total IgG and its subclasses in enzyme immunoassays; being used in this study as a marker of prior exposure to *P*. *vivax* infections. This protein is widely used in studies carried out in the Brazilian Amazon as one of the main markers of exposure to infection, being more antigenic than other parasite proteins [21]. The PvMSP1_19_ recombinant protein corresponding to the 19 kDa fragment of the C-terminal region (amino acids 1616–1704) of *P*. *vivax* MSP-1 was expressed in *Escherichia coli* as a fusion protein with glutathione S transferase (GST) of *Schistosoma japonicum*, as described in other studies [11,22,23] (**S2 Fig**). Unmodified recombinant GST was used as a control in the enzyme immunoassays for tests involving this protein.

### Enzyme immunoassay (ELISA) for detection of total IgG and subclasses

The total IgG response was measured against PvMSP1_19_ antigen by ELISA assays, according to the manufacturer’s protocols after standardizing the dilutions. All procedure details are described elsewhere [20]. For this assay, only the samples from the first collection/recruitment time-point were used. The measurements of absorbance values were performed using an automatic ELISA reader CLARIOstar Plus plate reader (BMG Labtech). All samples were performed in duplicate. Corrected absorbance values were obtained by subtracting absorbance readings with GST ran on the same microplate. Absorbance data used in statistical analyses correspond to corrected values. For quantification and classification of pregnant women, the Reactivity Index (RI) was used. RI was calculated as the ratio between the corrected absorbance values of each test sample and a cut-off value for antigen, corresponding to the average corrected absorbance for samples from 10 malaria-naïve blood donors plus 3 standard deviations. Positive samples had RIs levels greater than 1. Pregnant women were classified as non-exposed if the RI values was equal to or less than 1; and exposed if the level was greater than 1. In this way, the pregnant women were divided into four groups: non-infected–exposed and non-exposed; and *Pv*-infected–exposed and non-exposed to *P*. *vivax* infection before the current pregnancy.

Subclass detection was performed only on samples in which the total IgG was positive (RI>1). The procedure followed the same steps as for total IgG. However, in these subclass assays, monoclonal antibody binding was detected with biotinylated anti-rabbit immunoglobulin. Monoclonal antibodies specific for IgG 1 (clone RM117, Novus Biologicals), IgG 2 (clone #2348B, R&D Systems), IgG 3 (clone RM119, Novus Biologicals) and IgG 4 (clone RM120, Novus Biologicals) were used after standard dilution. Similar to total IgG, samples with RI levels greater than 1 were considered positive.

### Measurement of cytokines by cytometric bead array (CBA)

Quantification of cytokines was performed at different time-points of pregnancy, including at time of infections, in order to investigate the profile throughout the three gestational trimesters. The interleukin (IL-) 1β, IL-6, IL-8, IL10, IL-12p70, and TNF-α cytokines were detected and quantified in the maternal peripheral plasma by a CBA human inflammatory kit (BD Biosciences), according to the manufacturer’s protocol. Samples were analyzed in a two-laser BD FACSCalibur flow cytometer with CellQuest version 5.2 software (BD Biosciences), and concentrations computed using FCAP array software version 3.0.1 (BD Biosciences).

### Statistical analysis

For descriptive statistics, the variables were summarized using means and standard deviations, medians, and interquartile ranges, or using frequencies and percentages. Differences between groups were evaluated using Kruskal-Wallis test followed by the Dunn post-hoc multiple comparison test, Mann-Whitney test or T test when appropriate. Differences between categorical data and proportions were analyzed using Pearson′s chi-squared test. Associations between explanatory variables and covariates were also explored by regression models and correlation analysis with the aim of controlling for confounding factors. Two-tailed hypothesis tests at a significance level of 0.05 and 95% confidence intervals (CI) were estimated. For the calculation and exclusion of outliers, the Grubbs test was used (Alpha = 0.05) up to a maximum of 5% of exclusion in relation to the total N of each group. All analyses were performed using Stata/SE (v14.2), R (v4.2.2), and GraphPad Prism (v6.0) software.

## Results

### Baseline participant characteristics

600 pregnant participants were enrolled in the main study [19,20]. Two hundred and forty-two (40.3%) met the inclusion criteria for the present study. Of these, 143 (59.1%) were classified as non-infected (NI) and 99 (40.9%) pregnant women as being infected with *P*. *vivax* (*Pv*) during gestation (**S1 Fig**). The overall socio-demographic profile of the subjects stratified by group (NI and *Pv*) and by exposure based on the total IgG result, are shown in **Table 1**. Participants in the *Pv*-infected group differed significantly from non-infected participants by age (*P* = 0.008), educational level (*P* < 0.001), occupation (*P* = 0.007), and place of residence (*P* < 0.001) (**Table 1**). Regarding the place of residence, there is a similarity between the non-infected group and those who had *Pv* infection in their current pregnancy but do not have a history of malaria in their lifetime, in contrast to the group who have a history of *Pv* infection (3.5%, 5.9% *vs*. 26.8%). In addition, parasitemia was almost twice as high in the first infection in the *Pv*-exposed group (**Table 1**).

**Table 1:** Characteristics of the pregnant women according to previous exposure to the parasite.

Characteristics	Non-infected (N = 143)	*P*. *vivax* (N = 99)	*P* value^a^	*Pv* non-exposed (N = 17)	*Pv* exposed (N = 82)	*P* value^b^
Age in years	24.0 (19.0–29.0)	21.0 (17.0–26.0)	**0.008**	18.0 (16.0–26.0)	21.0 (17.0–26.0)	0.34
Gravidity	2.0 (1.0–3.0)	2.0 (1.0–3.0)	0.48	1.0 (1.0–2.0)	2.0 (1.0–3.0)	0.09
Education level, n (%)			**< 0.0001**			0.20
No education	0	2 (2.0)		1 (5.9)	1 (1.2)	
Primary	5 (3.5)	9 (9.1)		0	9 (11.0)	
≥ Secondary	138 (96.5)	88 (88.9)		16 (94.1)	72 (87.8)	
Occupation, n (%)			**0.007**			0.37
Farmer	3 (2.1)	7 (7.1)		0	7 (8.5)	
Housewife	66 (46.2)	50 (51.0)		11 (68.8)	39 (47.6)	
Student	29 (20.3)	27 (27.6)		3 (18.7)	24 (29.3)	
Other occupation	45 (31.4)	14 (14.3)		2 (12.5)	12 (14.6)	
Rural residence, n (%)	5 (3.5)	23 (23.2)	**< 0.0001**	1 (5.9)	22 (26.8)	0.06
Gestational age at 1^st^ infection in weeks	NA	21 (13.0–32.0)	NA	16.0 (12.0–24.0)	24.0 (13.0–32.0)	0.22
Trimester of 1^st^ infection, 1^st^/2^nd^/3^rd^ (%)	NA	27.3/35.4/37.3	NA	35.3/41.2/23.5	25.6/34.2/40.2	NA
Parasitemia at 1^st^ infection ^c^	NA	1094.5 (161.3–4175.0)	NA	715.6 (23.8–2140.0)	1218.0 (226.8–4421.0)	0.25

Abbreviations: N, total number of individuals; NA, not applicable. Results are presented as median and interquartile range or total number of events and percentage. Statistical tests were applied according to the type of variable (Mann-Whitney or chi-square).

^**a**^ Comparison between Non-infected and *P*. *vivax* infected groups.

^**b**^ Comparison between *P*. *vivax* non-exposed and *P*. *vivax* exposed according to total IgG to PvMSP1_19_.

^**c**^ Parasitemia in the 1^st^ infection was recorded in 94 pregnant women.

### Prior exposure to *P*. *vivax* alters the inflammatory profile in *Pv*-infection during pregnancy

**Fig 1:**
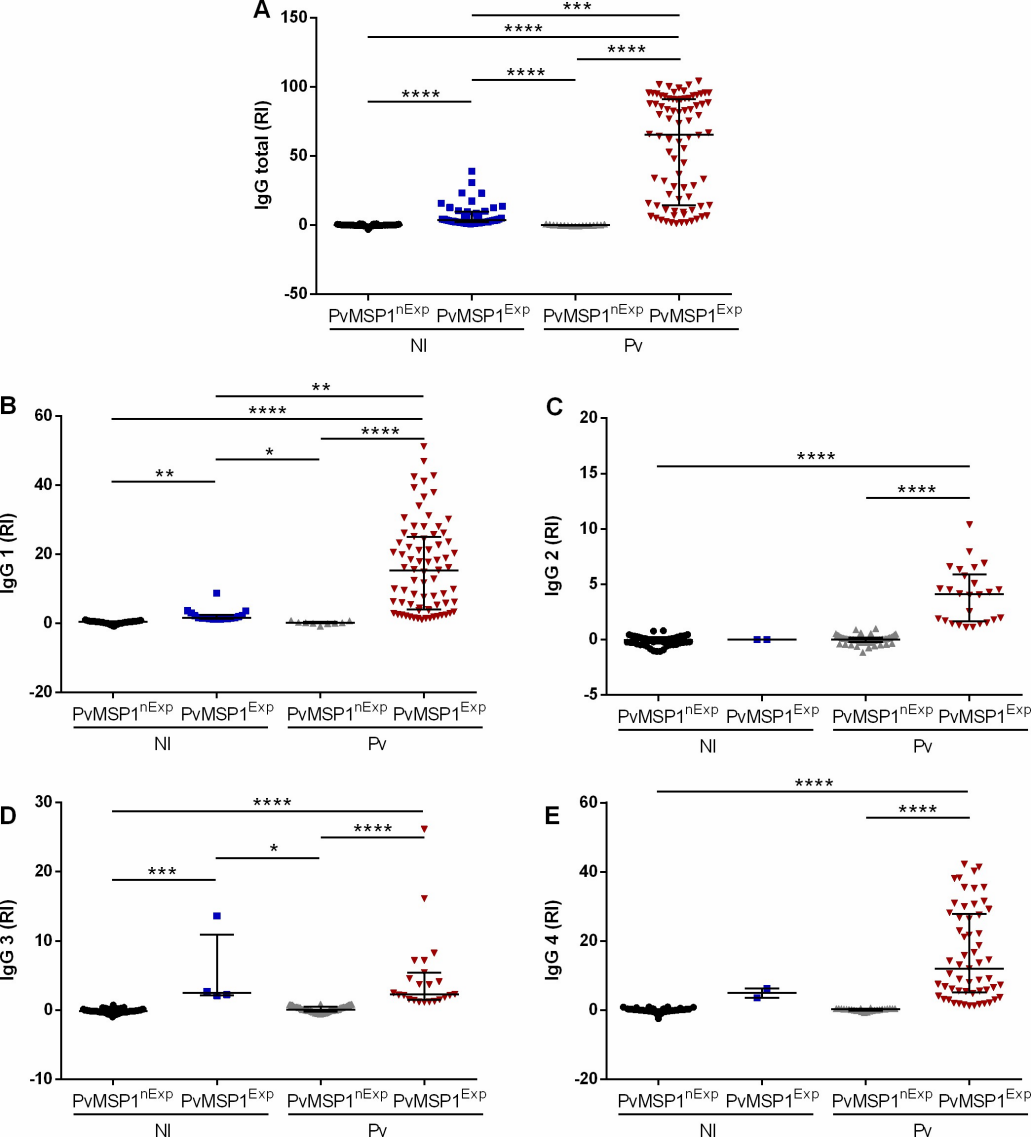
Antibody profile among non-infected and *P*. *vivax*-infected pregnant women. NI-PvMSP1^nExp^ -noninfected non-exposed (N = 39–97); NI-PvMSP1^Exp^—non-infected exposed (N = 2–45); Pv-PvMSP1^nExp^—*P*. *vivax*-infected non-exposed (N = 9–59); Pv-PvMSP1^Exp^—*P*. *vivax*-infected exposed (N = 23–82). PvMSP1- *Plasmodium vivax* merozoite surface protein 1. RI—Reactivity indices were calculated as the ratio between each test sample’s corrected absorbance values and a cut-off value for antigen, corresponding to the average corrected absorbance of samples from 10 malaria-naïve blood donors plus 3 standard deviations. Data are represented as scatter plot, with the median and the interquartile range. Group differences were evaluated by Kruskal-Wallis tests with Dunn’s post-test. *P < 0.05, **P < 0.01, ***P < 0.001, ****P < 0.0001.

Previous exposure to *P*. *vivax* was defined as an existent antibody response against PvMSP1_19_ at the first time-point of collection during pregnancy. When the pregnant woman had a history of *Pv*-malaria in life (before current pregnancy) and a *Pv*-infection during pregnancy, there was a significant increase in antibody levels (**Fig 1**).

**Fig 2:**
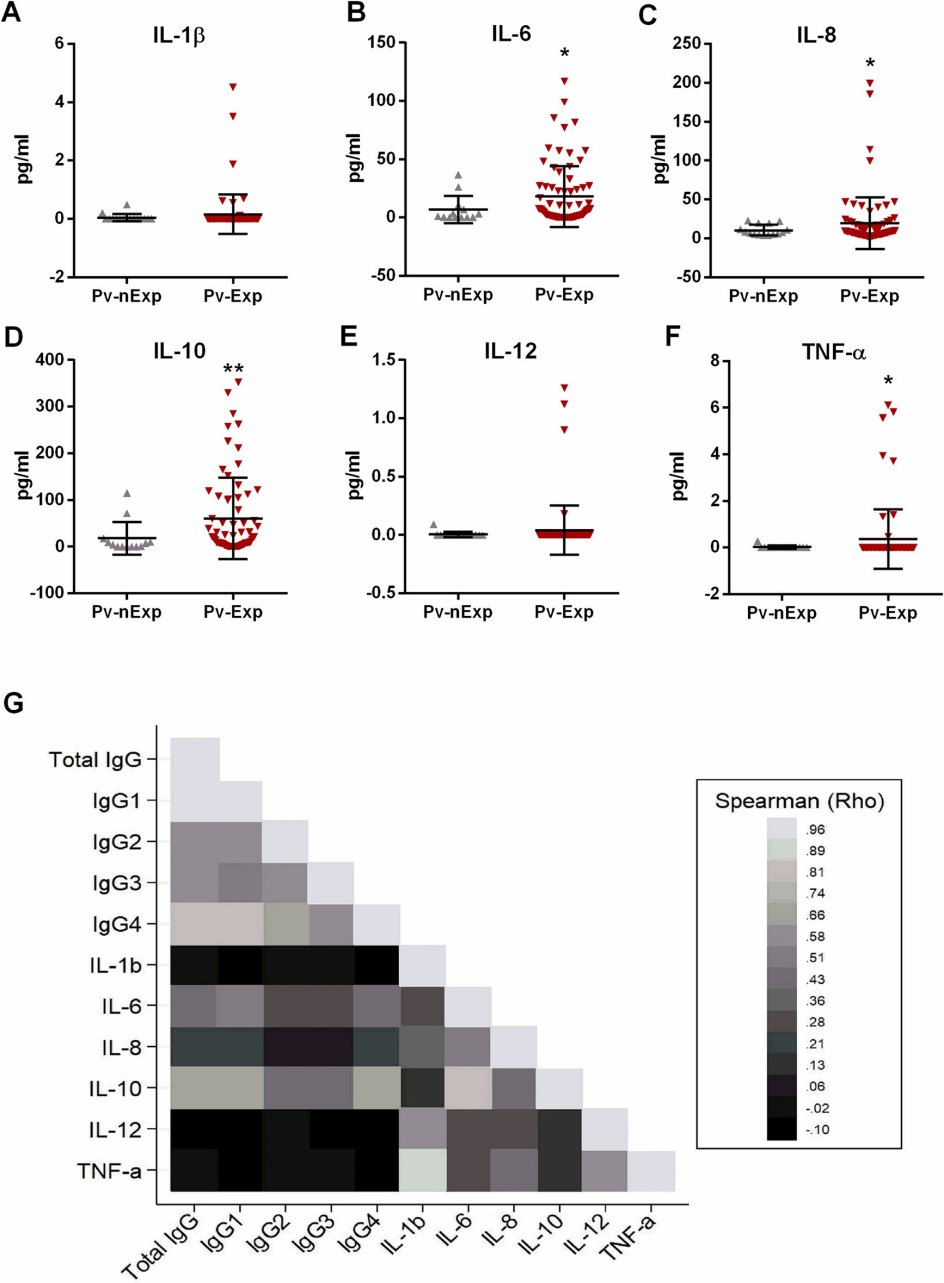
Screening of the inflammatory profile of *P*. *vivax-*infected pregnant women. Levels of cytokines in non-exposed (nExp) and exposed (Exp) pregnant women based on total IgG result for PvMSP1_19_ protein: (A-F) interleukin (IL)-1β, IL-6, IL-8, IL-10, IL-12, and TNF-α. (G) Heatmap of Spearman’s Rho correlation coefficients between antibody and inflammatory profiles. (A-F) Data are represented as mean and standard deviation and the t-test with Welch’s correction was used. (G) The colour bar on the right indicates Spearman’s Rho coefficient from no correlation (black) to positive correlation (light grey) and Bonferroni-adjusted test was used. *P < 0.05, **P < 0.01.

To assess whether previous exposure to malaria influences the inflammatory profile during infection, we quantified cytokines at their first infection in the current pregnancy (**Fig 2**). As a result, pregnant women with a history of malaria had high levels of all evaluated cytokines when compared to pregnant women without prior exposure to the parasite; this increase was more relevant among the cytokines IL-6, IL-8, IL-10 and TNF-α (**Fig 2B, 2C, 2D** and **2F**, respectively).

Cytokine levels were quantified among non-infected and *Pv*-infected pregnant women in all trimesters, including at time of infections. We observed differences only for the cytokines IL-6 and IL-10, which were significantly increased in *Pv*-infected pregnant women (IL-6: NI 4.86 *vs*. *Pv* 10.45, *P* < 0.001; IL-10: NI 0.72 *vs*. *Pv* 32.99, *P* < 0.001) (**S1 Table**).

Then, we evaluated only samples collected at the time of infection (**S2 Table**). We observed that all inflammatory factors were altered when comparing the results of pregnant women in the non-infected group (**S1 Table**), revealing that during infection all cytokines are at the highest levels. Noteworthy, similar to the previous analyses, the profile of IL-6 and IL-10 cytokines show the greatest changes during infection, especially when the infection occurs in the 1^st^ gestational trimester (**S2 Table**).

We further related the humoral immune profile with the peripheral cytokine response in the first infection. Overall, we observed a positive Spearman correlation between PvMSP1_19_ total IgG levels and the cytokines IL-6 (Rho = 0.46) and IL-10 (Rho = 0.63) (both *P* < 0.001); we also found a positive correlation between both cytokines and all IgG subclasses (IL-6: Rho IgG 1 = 0.47, Rho IgG 2 = 0.30, Rho IgG 3 = 0.29, and Rho IgG 4 = 0.47, *P* < 0.001; IL-10: Rho IgG 1 = 0.65, Rho IgG 2 = 0.41, Rho IgG 3 = 0.44, and Rho IgG 4 = 0.63, *P* < 0.001) (**Fig 2G**). In addition, for the *Pv*-infected group with prior exposure to the parasite, using a linear regression model adjusted for confounding variables (age at recruitment, gravidity, gestational age, and site of residence), we observed that total IgG levels were positively associated with IL-10 levels. Among the subclasses, IgG 1, IgG 2 ang IgG 4 levels were negatively associated with IL-6 concentration, and were positively associated with IL-10 concentration in maternal peripheral plasma at the first infection (**S3 Table**).

When we evaluated the association between the humoral immune response at the first infection and the inflammatory response throughout pregnancy, using all time-points (longitudinal evaluation), we observed a positive association, mainly between IL-10 and subclasses IgG 2 and 4 (*P* = 0.004 and *P* < 0.001, respectively) (**S4 Table**).

### Inflammatory response to *Pv*-infection is associated with adverse pregnancy outcomes

To assess whether this change in the maternal peripheral inflammatory profile could influence gestational outcomes, both for the mother and the newborn, correlation analyses were performed. Three maternal and three foetal characteristics (**Table 2**) were selected, considering the extreme importance of those in the context of gestational malaria by *P*. *vivax* [20]. Among maternal characteristics, it is observed that IL-6 has a positive correlation with previous exposure to malaria (Pearson, *r* = 0.21; *P* = 0.001) and with parasitemia (*r* = 0.45; *P* < 0.001), and a negative correlation with maternal weight gain (*r* = -0.14; *P* = 0.050); IL-8 also has a positive correlation with parasitemia (*r* = 0.32; *P* = 0.002). Another cytokine that proved to be relevant for maternal outcomes was IL-10, which also showed a positive correlation with previous exposure to malaria (*r* = 0.29) and parasitemia (*r* = 0.47) (both *P* < 0.001) and a negative correlation with the gain of maternal weight (*r* = -0.18; *P* = 0.010). In addition, the cytokines IL-6, IL-8, and IL-10 also proved to be important when the outcomes of newborns were analysed. It is observed that these cytokines were negatively correlated with gestational age at birth (IL-6, *r* = -0.38; IL-8, *r* = -0.25; IL-10, *r* = -0.49; *P* < 0.001) (**Table 2**), indicating that increased IL6 and IL-8 levels have a deleterious effect on pregnancy. It is presumed that the increase in IL-10 is a mechanism of autoregulation of the immune system in the face of this exacerbated increase in pro-inflammatory cytokines.

**Table 2:** Pearson’s correlation coefficient between maternal and foetal characteristics and peripheral cytokines at recruitment (1^st^ infection).

**Cytokines (pg/mL)**	**Previous exposure** **(IgGt—PvMSP-1)**		**Maternal weight gain**		**Parasitemia**	
	***r***	***P***	***r***	***P***	***r***	***P***
IL-1β	-0.089	0.17	-0.035	0.62	-0.064	0.54
IL-6	**0.208**	**0.001**	**-0.137**	**0.05**	**0.448**	**<0.0001**
IL-8	0.050	0.45	0.041	0.55	**0.319**	**0.002**
IL-10	**0.299**	**<0.0001**	**-0.177**	**0.01**	**0.474**	**<0.0001**
IL-12	-0.093	0.15	-0.038	0.59	-0.023	0.82
TNF-α	-0.087	0.18	-0.024	0.73	-0.049	0.64
	**Newborn weight**		**Length**		**Gestational age at delivery**	
	***r***	***P***	***r***	***P***	***r***	***P***
IL-1β	-0.043	0.52	0.079	0.24	-0.063	0.34
IL-6	-0.098	0.14	-0.076	0.26	**-0.382**	**<0.0001**
IL-8	0.030	0.65	0.049	0.47	**-0.251**	**0.0001**
IL-10	**-0.143**	**0.03**	**-0.135**	**0.04**	**-0.494**	**<0.0001**
IL-12	-0.035	0.60	0.092	0.17	-0.060	0.36
TNF-α	-0.043	0.51	0.087	0.19	-0.061	0.35

Abbreviations: IgG, Total Immunoglobulin G; IL, interleukin; TNF-α, tumor necrosis factor alpha. In bold are the statistically significant correlations.

## Discussion

In this study, we characterized the humoral immune response profile against the PvMSP1_19_ antigen in association with the inflammatory profile in *P*. *vivax* infections in pregnant women from the Brazilian region of Vale do Juruá (western Amazon). The PvMSP1_19_ antibody profile showed a prevalence of, predominantly, IgG 1 and IgG 4 subtypes. Additionally, the levels of the peripheral cytokines IL-6 and IL-10 were elevated in infected women, which were correlated with previous maternal exposure to malaria, increased parasitemia, and reduced maternal weight gain and the gestational age at delivery.

The antibody profile anti-PvMSP1_19_ presented in our results differs from other studies [18,24,25]. While we found a prevalence of IgG 1 and IgG 4 against the PvMSP1_19_ antigen in pregnant women, a predominance of cytophilic IgG 1 and IgG 3 subtypes have been reported in the general population [24]. This cytophilic profile is also observed in *P*. *falciparum* infections and is associated with protection against severe disease by promoting parasite clearance through complement activation and antibody-dependent cellular cytotoxicity [26]. Furthermore, this cytophilic antibody response is also seen against other proteins such as PvTRAP, in individuals from the Amazon basin, Brazil [25] and VAR2CSA in the plasma of pregnant women infected with *P*. *falciparum* in Benin [18]. In contrast, *P*. *vivax* infections are marked by lower levels of these protective antibodies, which correlates with its generally milder but more chronic disease course [27]. Non-cytophilic IgG2 and IgG4 antibodies are more common in persistent infections and are associated with poorer outcomes [27]. Overall, the more robust antibody response in *P*. *falciparum* likely contributes to the greater severity of infections compared with *P*. *vivax*, helping to explain the differing clinical manifestations between these two malaria species [27].

In this regard, our results suggest that there is a different pathway of immunomodulation in these pregnant women mediated by the infection and the exposition profile or, perhaps, another variable that might be interfering with this pattern. In recent studies, the cytokine IL-10 was shown to stimulate the production of IgG 4 during infections [28,29]. The increase in IgG 4 levels might be related to persistent antigen exposure [30], which may be due to recurrences of the infection due to pregnant women not taking primaquine.

We hypothesize that the humoral immune response in pregnant women exposed to *P*. *vivax* antigen would be related to the maternal inflammatory profile. We found an association between total IgG and the subclasses with IL-6 and IL-10 levels. A recent study found an association between lower levels of cytokines towards a tolerant immune state and previous exposure to malaria in adults [31]. These authors proposed that parasite control can be antibody-mediated via a cytophilic immune response by shaping cytokine levels towards the reduction of pro-inflammatory responses [31]. Contrary to that, the total IgG profile of our sample did not show a cytophilic profile. Possibly, this finding may explain the increase in parasitemia shown in our results. Also, we observed a positive association between increased IgG 4 subclass levels and high levels of all cytokines measured in infected pregnant women. In another study, it is proposed that the cytokine IL-10 can stimulate the production of IgG 4 during infections [28,29]; however, the role of IgG 4 in infectious diseases is unclear, as it may be associated with pathogenicity or protection in different disorders [17,28].

During gestational malaria, there is an increase in the production of inflammatory cytokines that contribute to the elimination of the parasite, increasing the phagocytic activity of macrophages [32]. However, this overproduction can compromise the pregnancy with serious consequences, especially for the foetus [32]. Here, we observed a significant increase only in the cytokine IL-6 and IL-10 levels, and even though the other cytokines did not significantly differ, these results are in agreement with other studies in the literature [33,34]. During normal pregnancies, the balance of inflammatory cytokines shifts to an anti-inflammatory profile to maintain pregnancy and avoid to the placental local inflammatory process [35,36]. Although the paradigm of this balance of the Th1/Th2 immune response is an oversimplification of the process, it has already been observed that intense Th1-type responses during pregnancy cause an increase in cytokines such as TNF-α, IL-10, IL-1β and IL-12 in peripheral and/or placental blood [33,34]. This fact is observed in cases of gestational malaria and is associated with adverse pregnancy effects such as maternal anaemia, spontaneous abortions, and premature births [34].

A parallel can also be drawn between the differences in the inflammatory profile between *P*. *vivax* and *P*. *falciparum* infections. IL-10, as an anti-inflammatory cytokine, plays an essential regulatory role in both species, with higher IL-10 levels in *P*. *vivax* contributing to a milder disease but facilitating chronic relapses [37]. Although we have not measured TGF-β, it is known that this cytokine also regulates both infections, particularly in modulating inflammation in *P*. *falciparum* [38]. These differences highlight the need for public health strategies tailored to the specific challenges posed by each parasite, especially during the gestational period.

In *P*. *vivax* infection, maternal plasmatic cytokine imbalance may have an impact on pregnancy outcomes, as indicated by the positive correlation between both IL-6 and IL-10 levels with previous exposure to malaria and parasitemia, as well as a negative correlation with gestational age at delivery. These findings are in line with the results presented in our previous study carried out in this same population, in which *P*. *vivax* infection was associated with prematurity [20]. In addition, IL-8 showed a positive correlation with parasitemia and negative correlation with gestational age at delivery. In fact, this proinflammatory cytokine have been positively associated with microscopic *P*. *vivax* infection in pregnant women [5].

IL-6 is a pro-inflammatory and pleiotropic cytokine with multiple functions, especially in the immune response to infections [39–41]. Elevated levels of this cytokine have also been observed in different conditions, including chronic diseases, and viral, bacterial, and parasitic infections, and may play a key role in mediating inflammation [40,42]. Also, it seems to be associated with increased severity of infections and infectious agents survival [41]. Therefore, IL-6 has been studied as a potential point of therapeutic intervention for several conditions [39].

IL-10 plays an essential role in regulating inflammation during malaria and tissue damage caused by TNF [43]. Imbalance of this cytokine has been linked to adverse events such as foetal loss weight, and an IL-10 knockout mouse model showed a link with preeclampsia, preterm birth, and foetal loss [44]. Our findings show an increase in IL-10 levels in the blood of pregnant women infected by *P*. *vivax*. A pro- and anti-inflammatory cytokine balance is important to provide protection and avoid complications [43]. By increasing IL-10 in *Pv*-infected pregnant women, there might be an attempt to return to a tolerant immune state by suppressing pro-inflammatory cytokines like IL-6 [31]. Our results also indicated a negative correlation between IL-10 and maternal gestational weight gain. A study in the Philippines found that the ratio of cytokines IL-6 and IL-10 was a reliable predictor of newborn weight and height [45], yet no correlation has been reported regarding maternal weight gain [46]. Both cytokines seem to affect the pathogenicity of malaria in pregnancy and its complications. However, the complexity of the role of IL-10 in malaria pathogenicity during pregnancy requires further study, as the relationship between this cytokine and newborn weight and height is still uncertain.

A comprehensive understanding of the distinct immune responses elicited by *P*. *vivax* and *P*. *falciparum* is essential for explaining the differences in disease severity and informing effective treatment strategies. This requires analyzing both cytokine profiles and immunoglobulin subclass responses, which provide valuable insights into how each parasite interacts with the host immune system and influences their respective clinical outcomes [47]. In *P*. *falciparum* infections, elevated levels of pro-inflammatory cytokines like TNF-α, IFN-γ, and IL-6 are critical for controlling the parasite. Still, they are also linked to severe complications such as cerebral malaria and anaemia [48]. In contrast, *P*. *vivax* induces lower levels of these cytokines, which results in fewer severe cases but can lead to chronic inflammation [47], similar to what we observed in this study in pregnant women.

Our work has some potential limitations. All blood samples were collected in heparin vacutainers; therefore, we cannot rule out contamination of plasma by platelet-derived factors. Furthermore, as our population is stratified into different groups for the analyses, the reduced number in some groups prevented us from performing more detailed analyses on the relationship between cytokines and malaria prospectively. It is also important to emphasize that we know the importance of several other cytokines such as IFN-γ and TGF-β for the immune response against the parasite, but unfortunately we did not have material available for further experiments. We therefore emphasize the importance of carrying out more prospective studies, with broader panels of cytokines and cellular components, to increase our knowledge of the pathogenesis of malaria in pregnant women. Lastly, although PvMSP1_19_ is widely used as a marker of previous exposure, it is not possible to confirm that all IgG-negative pregnant women have never been exposed to the parasite.

Finally, despite the high prevalence and important complications associated with the infection, there is a lack of longitudinal studies to better understand the consequences of gestational malaria by *P*. *vivax* in Latin America. Our results help to understand the association between anti-PvMSP1_19_ antibodies, considered exposure marker, and maternal peripheral inflammatory profile throughout pregnancy in the gestational malaria caused by *P*. *vivax*, bringing important implications for understanding the immunobiology of the disease.

## Supporting information

S1 Fig Flow diagram detailing exclusion criteria. (DOCX)

S2 Fig Proteins used for determination of antiPvMSP1_19_-specific antibodies by ELISA. (DOCX)

S1 Table Inflammatory factors in peripheral plasma of noninfected and infected pregnant women in all time-points during pregnancy. (DOCX)

S2 Table Inflammatory factors in peripheral plasma of *Pv*-infected pregnant women, only at the time of infection and by gestational trimester. (DOCX)

S3 Table Association of pro- and anti-inflammatory cytokines with immunoglobulins at first infection. (DOCX)

S4 Table Linear mixed-effect models for humoral immune response and cytokines. (DOCX)

S1 Data Study database. (XLSX)
