# Supplementary material for: Association of the humoral immune response with the inflammatory profile in Plasmodium vivax infections in pregnant women
Source: PLoS Negl Trop Dis. 2024 Nov 4;18(11):e0012636. doi: 10.1371/journal.pntd.0012636 (PMC11563365; doi:10.1371/journal.pntd.0012636)
Supplement: S1 Fig — (DOCX) [file pntd.0012636.s001.docx]

**86** **Mother-child pairs excluded**

| **52** | Alcohol and smoke |
| --- | --- |
| **30** | Other infectious diseases |
| **1** | Pre-eclampsia |
| **3** | >1 foetus or congenital anomaly |

**272** **Mother-child pairs excluded**

| **40** | Undefined gestational age |
| --- | --- |
| **72** | Mixed-infected |
| **73** | *P. falciparum*-infected |
| **44** | *P. vivax*-infected (No sample from 1^st^ infection) |
| **43** | Protozoa and helminths |

**514** Mother-child pairs initially included

**600** **Mother-child pairs**

[Delivered 01/2013 - 04/2015]

**242** **Mother-child pairs included in the analysis**

| **143** | Non-infected mothers |
| --- | --- |
| **99** | *P. vivax*-infected mothers |

**S1 Fig.** **Flow diagram detailing exclusion criteria.** Mixed infection – *P. vivax*- and *P. falciparum*-infection occurring at the same time and at different times during pregnancy. Exclusions were made to avoid misinterpretation of the results since several other factors can interfere with gestational outcomes.
