# Supplementary material for: Association of the humoral immune response with the inflammatory profile in Plasmodium vivax infections in pregnant women
Source: PLoS Negl Trop Dis. 2024 Nov 4;18(11):e0012636. doi: 10.1371/journal.pntd.0012636 (PMC11563365; doi:10.1371/journal.pntd.0012636)
Supplement: S2 Fig — (DOCX) [file pntd.0012636.s002.docx]

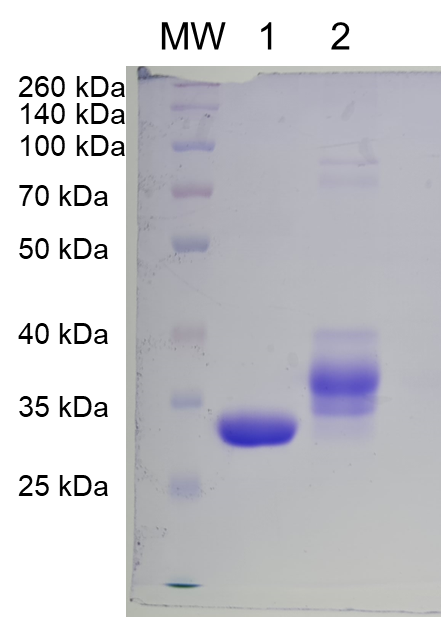


**S2 Fig. Proteins used for determination of antiPvMSP1_19_-specific antibodies by ELISA.** MW is Broad Range Molecular Weight Standard (ThermoScientific). Lane 1 contains 5 µg unfused GST and lane 2 5 µg PvMSP119 C-terminally fused to GST, produced in *E. coli* BL21 pGRO7 bacterial cells and purified in batch using Glutathione sepharose (GE Healthcare) as described in de Oliveira et al. 1999*. Proteins were separated under non-reducing conditions (without DTT or mercaptoethanol) in a 10% SDS-polyacrylamide gel, and stained with Coomassie blue. GST-PVMSP119 may contain truncated and multimeric forms (faint bands at ~80 kDa).

*(Vaccine. 1999 Aug 6;17(23-24):2959-68. doi: 10.1016/s0264-410x(99)00176-0.)
