# Supplementary material for: Association of the humoral immune response with the inflammatory profile in Plasmodium vivax infections in pregnant women
Source: PLoS Negl Trop Dis. 2024 Nov 4;18(11):e0012636. doi: 10.1371/journal.pntd.0012636 (PMC11563365; doi:10.1371/journal.pntd.0012636)
Supplement: S1 Table — (DOCX) [file pntd.0012636.s003.docx]

**S1 Table. Inflammatory factors in peripheral plasma of noninfected and infected pregnant women in all time-points during pregnancy.**

| **Cytokines (pg/mL)** | **Non-infected**  **N=143 (490 samples)** | ***P. vivax***  **N=99 (330 samples)** | ***P* value** |
| --- | --- | --- | --- |
| IL-1β | 0.41 ± 1.73 | 0.57 ± 3.20 | 0.99 |
| IL-6 | 4.86 ± 11.03 | 10.45 ± 20.13 | < 0.0001 |
| IL-8 | 15.43 ± 21.09 | 15.99 ± 21.65 | 0.17 |
| IL-10 | 0.72 ± 2.87 | 32.99 ± 80.68 | < 0.0001 |
| IL-12 | 0.17 ± 0.84 | 0.04 ± 0.24 | 0.27 |
| TNF-α | 0.65 ± 2.45 | 0.48 ± 1.90 | 0.51 |

Abbreviations: IL, interleukin; TNF-α, tumor necrosis factor alpha. Results are presented as mean and standard deviation. Differences between groups were determined by the Mann-Whitney test.

IL-1 – exclusion 6 samples NI (values 28.34-146.73) + 4 samples Pv (values 32.70-164.56) outliers

IL-6 – exclusion 4 samples NI (values 145.36-175.84) + 14 samples Pv (values 139.71-10165.58) outliers

IL-8 – exclusion 18 samples NI (values 121.10-718.97) + 8 samples Pv (values 185.14-592.95) outliers

IL-10 – exclusion 5 samples NI (values 42.98-274.74) + 17 samples Pv (values 472.12-10832.36) outliers

IL-12 – exclusion 9 samples NI (values 9.55-130.94) + 1 sample Pv (value 81.34) outlier

TNF-α – exclusion 10 samples NI (values 26.13-232.43) + 9 samples Pv (values 22.18-149.23) outliers
