# Supplementary material for: Association of the humoral immune response with the inflammatory profile in Plasmodium vivax infections in pregnant women
Source: PLoS Negl Trop Dis. 2024 Nov 4;18(11):e0012636. doi: 10.1371/journal.pntd.0012636 (PMC11563365; doi:10.1371/journal.pntd.0012636)
Supplement: S2 Table — (DOCX) [file pntd.0012636.s004.docx]

**S2 Table. Inflammatory factors in peripheral plasma of *Pv*-infected pregnant women, only at the time of infection and by gestational trimester.**

| **Cytokines (pg/mL)** | **Total positive samples**  **(n=170)** | ***P. vivax***  **N=99 pregnant women - 170 positive samples** | | |
| --- | --- | --- | --- | --- |
|  |  | **1^st^ Tri**  **(n=27)** | **2^nd^ Tri**  **(n=58)** | **3^rd^ Tri**  **(n=85)** |
| IL-1β | 0.83 ± 4.20 | 0.08 ± 0.20 | 1.87 ± 6.63 | 0.37 ± 2.09 |
| IL-6 | 29.31 ± 65.93 | 28.78 ± 55.45 | 12.86 ± 21.11 | 18.89 ± 26.96 |
| IL-8 | 22.76 ± 36.64 | 22.00 ± 28.75 | 13.77 ± 11.35 | 20.56 ± 28.37 |
| IL-10 | 83.31 ± 142.70 | 100.1 ± 171.90 | 78.76 ± 121.50 | 67.22 ± 116.7 |
| IL-12 | 0.05 ± 0.29 | 0.03 ± 0.11 | 0.05 ± 0.21 | 0.07 ± 0.37 |
| TNF-α | 1.30 ± 5.53 | 0.56 ± 2.16 | 2.69 ± 8.88 | 0.58 ± 2.11 |

Abbreviations: IL, interleukin; TNF-α, tumor necrosis factor alpha. Results are presented as mean and standard deviation. 45 pregnant women had 2 or more infections during pregnancy.
