# Supplementary material for: Association of the humoral immune response with the inflammatory profile in Plasmodium vivax infections in pregnant women
Source: PLoS Negl Trop Dis. 2024 Nov 4;18(11):e0012636. doi: 10.1371/journal.pntd.0012636 (PMC11563365; doi:10.1371/journal.pntd.0012636)
Supplement: S3 Table — (DOCX) [file pntd.0012636.s005.docx]

**S3 Table. Association of pro- and anti-inflammatory cytokines with immunoglobulins at first infection.**

|  | **Total IgG** | **IgG 1** | **IgG 2** | **IgG 3** | **IgG 4** |
| --- | --- | --- | --- | --- | --- |
|  | **Coef.**  **(95% CI)** | **Coef.**  **(95% CI)** | **Coef.**  **(95% CI)** | **Coef.**  **(95% CI)** | **Coef.**  **(95% CI)** |
| IL-1β | -0.78  (-10.57; 9.00) | 0.12  (-3.28; 3.51) | 0.16  (-0.44; 0.76) | 0.03  (-2.60; 2.67) | 0.22  (-3.10; 3.55) |
| IL-6 | -0.01  (-0.03; 0.001) | **-0.01**  **(-0.02; -0.004)** | -**0.002**  **(-0.003; -0.001)** | -0.001  (-0.001; 0.004) | **-0.01**  **(-0.01; -0.002)** |
| IL-8 | -0.09  (-0.36; 0.19) | -0.03  (-0.13; 0.06) | 0.00  (-0.02; 0.02) | -0.01  (-0.09; 0.06) | -0.01  (-0.10; 0.09) |
| IL-10 | **0.02**  **(0.005; 0.04)** | **0.01**  **(0.01; 0.02)** | **0.002**  **(0.001; 0.003)** | 0.001  (-0.004; 0.01) | **0.01**  **(0.01; 0.02)** |
| IL-12 | -0.15  (-2.26; 1.96) | -0.31  (-1.05; 0.42) | -0.04  (-0.17; 0.09) | 0.02  (-0.55; 0.59) | 0.15  (-0.57; 0.87) |
| TNF-α | -0.23  (-7.48; 7.03) | -0.27  (-2.78; 2.25) | -0.14  (-0.59; 0.30) | -0.05  (-2.01; 1.90) | -0.38  (-2.85; 2.08) |

Abbreviations: IgG, Total Immunoglobulin G; IL, interleukin; TNF-α, tumor necrosis factor alpha. Multivariable linear regression models adjusting for the following variables: age at recruitment, gravidity, gestational age, and site of residence. N = 82. In bold if 95% confidence interval (CI) does not include 0. In bold are the statistically significant correlations (*P* < 0.05).
