# Supplementary material for: Association of the humoral immune response with the inflammatory profile in Plasmodium vivax infections in pregnant women
Source: PLoS Negl Trop Dis. 2024 Nov 4;18(11):e0012636. doi: 10.1371/journal.pntd.0012636 (PMC11563365; doi:10.1371/journal.pntd.0012636)
Supplement: S4 Table — (DOCX) [file pntd.0012636.s006.docx]

**S4 Table. Linear mixed-effect models** **for humoral immune response and cytokines.**

| **Fitting models** | **Estimate** | **Standard error** | ***P value*** |
| --- | --- | --- | --- |
| **TOTAL IgG** |  |  |  |
| Intercept | 0.583 | 0.172 | 0.001 |
| IL-12 | -0.001 | 0.003 | 0.62 |
| Intercept | 0.582 | 0.172 | 0.001 |
| TNF-α | -0.001 | 0.001 | 0.46 |
| Intercept | 0.602 | 0.171 | 0.0006 |
| IL-10 | <0.001 | <0.001 | 0.13 |
| Intercept | 0.605 | 0.172 | 0.0006 |
| IL-6 | <0.001 | <0.001 | 0.15 |
| Intercept | 0.584 | 0.172 | 0.001 |
| IL-1β | -0.002 | 0.002 | 0.41 |
| Intercept | 0.610 | 0.172 | 0.0005 |
| IL-8 | <0.001 | <0.001 | 0.19 |
|  |  |  |  |
| **IgG 1** |  |  |  |
| Intercept | 0.144 | 0.185 | 0.44 |
| IL-12 | -0.002 | 0.003 | 0.50 |
| Intercept | 0.145 | 0.184 | 0.43 |
| TNF-α | -0.001 | 0.001 | 0.38 |
| Intercept | 0.164 | 0.182 | 0.37 |
| IL-10 | <0.001 | <0.001 | 0.06 |
| Intercept | 0.160 | 0.184 | 0.39 |
| IL-6 | <0.001 | <0.001 | 0.22 |
| Intercept | 0.148 | 0.184 | 0.42 |
| IL-1β | -0.002 | 0.002 | 0.28 |
| Intercept | 0.176 | 0.184 | 0.34 |
| IL-8 | 0.001 | 0.001 | 0.10 |
|  |  |  |  |
| **IgG 2** |  |  |  |
| Intercept | 0.190 | 0.108 | 0.08 |
| IL-12 | 0.001 | 0.002 | 0.71 |
| Intercept | 0.190 | 0.108 | 0.08 |
| TNF-α | <0.001 | <0.001 | 0.79 |
| Intercept | 0.210 | 0.105 | 0.05 |
| IL-10 | **<0.001** | **<0.001** | **0.004** |
| Intercept | 0.206 | 0.107 | 0.06 |
| IL-6 | <0.001 | <0.001 | 0.06 |
| Intercept | 0.190 | 0.108 | 0.08 |
| IL-1β | <0.001 | <0.001 | 0.87 |
| Intercept | 0.213 | 0.107 | 0.05 |
| IL-8 | **<0.001** | **<0.001** | **0.05** |
|  |  |  |  |
| **IgG 3** |  |  |  |
| Intercept | -0.198 | 0.171 | 0.25 |
| IL-12 | <0.001 | 0.002 | 0.95 |
| Intercept | -0.198 | 0.171 | 0.25 |
| TNF-α | <0.0001 | 0.001 | 0.99 |
| Intercept | -0.180 | 0.169 | 0.29 |
| IL-10 | <0.001 | <0.001 | 0.10 |
| Intercept | -0.180 | 0.170 | 0.29 |
| IL-6 | <0.001 | <0.001 | 0.18 |
| Intercept | -0.198 | 0.171 | 0.15 |
| IL-1β | <0.001 | 0.002 | 0.96 |
| Intercept | -0.176 | 0.171 | 0.30 |
| IL-8 | <0.001 | <0.001 | 0.26 |
|  |  |  |  |
| **IgG 4** |  |  |  |
| Intercept | 0.447 | 0.162 | 0.007 |
| IL-12 | -0.002 | 0.002 | 0.31 |
| Intercept | 0.449 | 0.162 | 0.007 |
| TNF-α | -0.002 | 0.001 | 0.18 |
| Intercept | 0.480 | 0.154 | 0.002 |
| IL-10 | **<0.001** | **<0.001** | **0.0003** |
| Intercept | 0.475 | 0.159 | 0.004 |
| IL-6 | **<0.001** | **<0.001** | **0.02** |
| Intercept | 0.451 | 0.162 | 0.006 |
| IL-1β | -0.003 | 0.002 | 0.18 |
| Intercept | 0.490 | 0.159 | 0.003 |
| IL-8 | **0.001** | **<0.001** | **0.01** |

Other parameters included in each of the models: infection group, number of infections during pregnancy, maternal age, place of residence and number of malaria cases before pregnancy. In bold are the statistically significant associations of cytokines.

Abbreviations: IL, interleukin; TNF-α, tumor necrosis factor alpha.
